# Lipid peroxidation and glutathione peroxidase activity relationship in breast cancer depends on functional polymorphism of *GPX1*

**DOI:** 10.1186/s12885-015-1680-4

**Published:** 2015-10-07

**Authors:** Ewa Jablonska, Jolanta Gromadzinska, Beata Peplonska, Wojciech Fendler, Edyta Reszka, Magdalena B. Krol, Edyta Wieczorek, Agnieszka Bukowska, Peter Gresner, Michal Galicki, Oskar Zambrano Quispe, Zbigniew Morawiec, Wojciech Wasowicz

**Affiliations:** Department of Toxicology and Carcinogenesis, Nofer Institute of Occupational Medicine, 8 Sw. Teresy Str, Lodz, Poland; Department of Environmental Epidemiology, Nofer Institute of Occupational Medicine, 8 Sw. Teresy Str, Lodz, Poland; Department of Pediatrics, Oncology, Hematology and Diabetology, Medical University of Lodz, 36/50 Sporna Str, Lodz, Poland; Department of Surgical Oncology, Regional Cancer Center, Copernicus Memorial Hospital in Lodz, 62 Pabianicka Str, Lodz, Poland

**Keywords:** Breast cancer, Lipid peroxidation, Glutathione peroxidase, *GPX1*, Single nucleotide polymorphism, Selenium

## Abstract

**Background:**

Since targeting oxidative stress markers has been recently recognized as a novel therapeutic target in cancer, it is interesting to investigate whether genetic susceptibility may modify oxidative stress response in cancer. The aim of this study was to elucidate whether genetic polymorphism in the antioxidant enzymes is associated with lipid peroxidation in breast cancer.

**Methods:**

We conducted a study among Polish women, including 136 breast cancer cases and 183 healthy controls. The analysis included genetic polymorphisms in five redox related genes: *GPX1* (rs1050450), *GPX4* (rs713041), *SOD2* (rs4880), *SEPP1* (rs3877899) and *SEP15* (rs5859), lipid peroxidation, the activities of antioxidant enzymes determined in blood compartments as well as plasma concentration of selenium – an antioxidant trace element involved in cancer. Genotyping was performed using the Real Time PCR. Lipid peroxidation was expressed as plasma concentration of thiobarbituric acid reactive substances (TBARS) and measured with the spectrofluorometric method. Glutathione peroxidase activity was spectrophotometrically determined in erythrocytes (GPx1) and plasma (GPx3) by the use of Paglia and Valentine method. Spectrophotometric methods were employed to measure activity of cytosolic superoxide dismutase (SOD1) in erythrocytes (Beauchamp and Fridovich method) and ceruloplasmin (Cp) in plasma (Sunderman and Nomoto method). Plasma selenium concentration was determined using graphite furnace atomic absorption spectrophotometry.

**Results:**

Breast cancer risk was significantly associated with *GPX1* rs1050450 (Pro198Leu) polymorphism, showing a protective effect of variant (Leu) allele. As compared to the control subjects, lipid peroxidation and GPx1 activity were significantly higher in the breast cancer cases, whereas ceruloplasmin activity was decreased. After genotype stratification, both GPx1 activity and TBARS concentration were the highest in *GPX1* Pro/Pro homozygotes affected by breast cancer. At the same time, there was a significant correlation between the level of lipid peroxidation and GPx1 activity among the cancer subjects possessing *GPX1* Pro/Pro genotype (*r* = 0.3043; *p* = 0.0089), whereas such a correlation was completely absent in the cases carrying at least one *GPX1* Leu allele as well as in the controls (regardless of *GPX1* genotype).

**Conclusions:**

*GPX1* polymorphism may be an important factor modifying oxidative stress response in breast cancer subjects. Further studies are needed to elucidate its potential clinical significance.

**Electronic supplementary material:**

The online version of this article (doi:10.1186/s12885-015-1680-4) contains supplementary material, which is available to authorized users.

## Background

Breast cancer is a multifactorial and a complex disease, with a major etiological contribution of hormonal origin and about 5–10 % of risk attributable to the inherited genetic factors (mainly associated with *BRCA1* and *BRCA2* mutations) [1]. Genetic variations associated with sporadic breast cancer as well as their interactions with environmental factors are still poorly understood. Similarly, pathological processes linked to breast cancer tissue are not entirely explored, though they are generally associated with oxidative stress [2]. Prooxidant processes in breast tissue are mainly linked to lipid peroxidation, as mammary gland is profusely surrounded by adipose tissue [2]. Notably, targeting oxidative stress markers has been recently recognized as a novel therapeutic approach in cancer treatment, due to the fact that generation of reactive oxygen species (ROS) as well as some products of lipid peroxidation may improve effectiveness of the treatment by decreasing cancer progression and reducing drug resistance. Mechanisms underlying these effects (and reviewed recently by Barrera [3]) are mainly associated with the induction of apoptosis in cancer cells by overcoming their antioxidant defense. The upregulated antioxidant defense is an extremely important adaptive mechanism in cancer cells, as it allows them to survive under conditions of permanent oxidative stress, and it is often associated with cancer progression and drug resistance. Thus targeting ROS has been suggested as a potential determinant of effective treatment in cancer [4, 5].

Since breast cancer is largely associated with lipid peroxidation, it may be hypothesized that the disease progression or response to treatment may highly rely on patient’s individual ability to scavenge either lipid peroxidation products or reactive species that lead to lipid oxidation (like hydroxyl radical). The interesting issue to be explored under this approach is whether genetic susceptibility associated with antioxidant system, may modify the prooxidative effects in breast cancer subjects. It is well known, that some genetic variations present in the antioxidant enzymes modify their activity or function, which may result in the altered ability to scavenge ROS [6]. These alterations explain some associations between specific gene variants and breast cancer risk [7–11], suggesting protective role of variants linked to the increased antioxidant protection. However, when the tumor is already developed, upregulated antioxidant system may act in an opposite way, promoting cancer cells growth and metastasis [12]. One may hypothesize that genetically determined high ability to scavenge reactive species and especially lipid peroxidation products, may serve as a negative prognostic factor in breast cancer subjects.

Natural antioxidant defense consists of many enzymatic and nonenzymatic systems that act in concert with dietary antioxidants [12]. Most important antioxidant enzymes include superoxide dismutases (SOD), glutathione peroxidases (GPx) and catalase (Cat). SOD (including 3 forms: cytosolic - SOD1, mitochondrial - SOD2 and extracellular - SOD3) catalyze dismutation of superoxide anion into hydrogen peroxide, whereas Cat and GPxs reduce hydrogen peroxide, thus preventing production of highly toxic hydroxyl radical [13]. Importantly, GPxs may also reduce hydroperoxides of polyunsaturated fatty acids, counteracting toxic effects of lipid peroxidation. Nonenzymatic endogenous antioxidants (apart from thiols) include metal-binding proteins which sequester prooxidant metals such as iron and copper [12]. One of the important metal-binding proteins is ceruloplasmin (Cp). This enzymatic protein binds copper ions (reducing their deleterious effects) and protects membrane lipids from iron-dependent lipid peroxidation due to its ferroxidase-type activity [13].

Endogenous antioxidant system is supported by exogenous factors derived from diet (like vitamins and trace elements) and the element which probably gained most of scientific interest in terms of its antioxidant properties, is selenium (Se). Many experimental and epidemiological findings suggest significant role of Se in cancer, notably both in its prevention and promotion, though neither one nor the other mechanism is yet fully understood [14, 15]. It is proposed that Se acts both via low molecular Se compounds and via specific proteins, called selenoproteins. Most of these proteins possess redox activity like for example already mentioned glutathione peroxidases, including GPx1 (cytosolic glutathione peroxidase), GPx2 (gastrointestinal glutathione peroxidase), GPx3 (plasma glutathione peroxidase), GPx4 (phospholipid hydroperoxide glutathione peroxidase) and GPx6 (olfactory glutathione peroxidase) [14]. The activity of GPxs largely depend on Se due to its presence at the active site of these enzymes [16]. There are also other physiologically important selenoproteins, like selenoprotein P (SelP), which is responsible for Se transport or selenoprotein 15 kDa (Sep15), which is involved in protein folding in endoplasmic reticulum [14].

The aim of this study was to investigate the overall relationship between lipid peroxidation, markers of antioxidant system and individual genetic susceptibility linked to antioxidant response in breast cancer subjects. Lipid peroxidation was measured as plasma concentration of thiobarbituric acid-reactive substances (TBARS). Markers of antioxidant system comprised the activity of the antioxidant enzymes in blood compartments (GPx1, GPx3, Cp and SOD1) and plasma concentration of Se. Polymorphic genes (Additional file 1: Table S1) covered: *GPX1*, *GPX4*, *SEPP1*, *SEP15* (all encoding selenoproteins) and *SOD2* (encoding SOD2).

## Materials and methods

### Study group

The study involved 136 cases and 183 women assigned to the control group. All the subjects were enrolled for the study in the years 2007–2012. The cases were female patients of the Copernicus Memorial Hospital in Lodz, Poland, diagnosed with a primary breast cancer. Basic epidemiological characteristics (age, BMI, smoking status and menopausal status) were collected using individual questionnaires, whereas clinical data (histological type of tumor, tumor stage and grade, receptor status, treatment) were obtained from medical records. The controls were selected from the population of the cross sectional study of nurses and midwives (registered at the Local Registry of the Chamber of Nurses and Midwifes in Lodz) who underwent mammography screening in the course of another study [17]. A detailed description of mammography density assessment was presented elsewhere [17]. On the basis of mammograms, the women who were reported to have a mass, distorted architecture, density or calcification in the breast tissue were excluded from the study (from the control group). The second selection criterion was based on the type of work with respect to shifts. Specifically, the women who were reported to work in shifts (at the time of recruitment) were not included in the study, because this factor was shown to affect the antioxidant status in the group [18]. A signed informed consent was obtained from all the participants and the study was conducted in compliance with the Declaration of Helsinki and with approval by the Local Ethics Committee (Ethical Institutional Review Board at the Nofer Institute of Occupational Medicine, Lodz, Poland, Resolution No 5/2007). Characteristics of the study groups is presented in Table 1.

**Table 1:** Characteristics of the study group

	Cases	Controls	*p*
N	136	183	
Age (years)	51.9 ± 6.5 (35–61)	51.4 ± 4.9 (40–60)	0.092^a^
BMI (kg/m^2^)	26.7 ± 4.8 (17.1**–**43.1)	27.2 ± 4.8 (18.6**–**48.3)	0.611^a^
Smoking status, n (%)			
Ever smokers	82 (60)	110 (60)	0.973^b^
Never smokers	54 (40)	73 (40)	
Current smoking, n (%)			
Yes	30 (22)	50 (27)	0.233^b^
No	106 (88)	133 (73)	
Menopausal status (self-reported), n (%)			
Postmenopausal	73 (54)	107 (58)	0.944^b^
Premenopausal	51 (37)	76 (42)	
Unknown	12 (9)	-	
Histological type, n (%)			
IDC	109 (80)	na	-
ILC	4 (3)		
DCIS	2 (1)		
LCIS	1 (1)		
Unknown	20 (15)		
Tumor stage, n (%)			
Tis	3 (2.2)	na	-
T1	60 (44.1)		
T2	55 (40.5)		
T3	2 (1.5)		
T4	3 (2.2)		
Tx	3 (2.2)		
Unknown	10 (7.3)		
Tumor grade, n (%)			
G1	8 (5.9)	na	-
G2	44 (32.4)		
G3	52 (38.2)		
Gx	15 (11.0)		
Unknown	17 (12.5)		
ER status, n (%)			
ER-positive	82 (60)	na	-
ER-negative	32 (24)		
Unknown	22 (16)		
PR status, n (%)			
PR-positive	78 (57.4)	na	-
PR-negative	44 (32.3)		
Unknown	14 (10.3)		
HER2 status, n (%)			
HER2-positive	19 (14.0)	na	-
HER2-negative	94 (69.1)		
Unknown	23 (16.9)		
Treatment, n (%)			
Yes^c^	21 (15)	na	-
No	115 (85)		

Data for age and BMI expressed as mean ± standard deviation (range)

*IDC* invasive ductal carcinoma, *ILC* invasive lobular carcinoma, *DCIS* ductal carcinoma *in situ*, *LCIS* lobular carcinoma *in situ*, *ER* estrogen receptors, *PR* progesterone receptors, *HER2* human epidermal growth factor receptors, *T* tumor stage, *G* tumor grade, *na* not applicable

^a^the Mann–Whitney test

^b^the Chi-squared test

^c^patients who underwent chemotherapy or breast cancer surgery

### Methods

Blood samples (7.5 mL) were collected into heparinized test tubes free from trace elements and separated by centrifugation into buffy coat (for DNA isolation), plasma and erythrocytes. Each fraction was stored at – 20 °C until analysis. Before freezing, erythrocytes were washed three times in isotonic saline and hemolysates were prepared followed freezing and thawing two times.

#### DNA isolation

DNA was isolated from buffy coat, using the QIAamp DNA Blood Mini Kit (Qiagen, Hilden, Germany) according to the manufacturer’s instructions. DNA purity and quantity were determined with a spectrophotometer (Eppendorf, Hamburg, Germany) at a wave length of 260 and 280 nm.

#### SNP genotyping

Allelic discrimination was performed using the Real Time PCR method and the CFX96™ Real Time PCR Detection System (Bio-Rad, Hercules, CA, USA). For genes: *GPX4* (rs713041), *SEPP1* (rs3877899) and *SOD2* (rs4880), we identified SNPs using Taqman® SNP Genotyping Assays (C_2561693_20, C_8709053_10 and C_2841533_10) and Taqman Genotyping Master Mix (Life Technologies, Carlsbad, CA, USA). PCR reactions were carried out with 10 ng of DNA in a final volume of 10 μL, under following conditions: 10 min at 95 °C enzyme activation and 50 two-step cycles of denaturation at 95 °C for 15 s and annealing at 60 °C for 1 min. For genes: *GPX1* (rs1050450) and *SEP15* (rs5859), we employed the High Resolution Melt Curve technique. Oligonucleotide sequences for PCR primers, designed by Beacon Designer™ (PREMIER Biosoft, Palo Alto, CA, USA), were as follows: 5′-GCCGCTTCCAGACCATTG-3′ (forward) and 5′-GGTGTTCCTCCCTCGTAG-3′ (reverse) for *GPX1*, 5′-TTGCGTTAATGAAGACTACACAG-3′ (forward) and 5′-AAACATGAAAGAACAAACCAGAAG-3′ (reverse) for *SEP15*. The Real-time PCR was performed in 20 μL volume, in the presence of 20 ng of genomic DNA, primers (0.5 μM each each), SsoFast™ EvaGreen® Supermix (Bio-Rad, Hercules, CA, USA) and nuclease-free water. The reaction protocol for both genes included enzyme activation at 98 °C for 3 min, followed by 40 two-step cycles of denaturation at 98 °C for 5 s and annealing at 57 °C (for *GPX1*) or 60 °C (for *SEP15*) for 10 s. The protocol for melting curve analysis, performed immediately after the PCR, included initial DNA denaturation at 95 °C for 1 min, followed by 150 two-step cycles: DNA renaturation at 65 °C for 1 min and DNA denaturation with the 0.2 temperature increment in each cycle (from 65 °C to 95 °C in the last cycle). Data analysis was performed using the Bio-Rad CFX Manager and the Bio-Rad Precision Melt Analysis Software. Particular genotypes for *GPX1* and *SEP15* were identified on the basis of PCR-RFLP method, using following restriction enzymes: *DDe*I (Promega, Madison, WI, USA) fo *GPX1* and *FspB*I (Fermentas, Waltham, MA, USA) for *SEP15*. Oligonucleotide sequences for PCR primers were: GPX1 forward 5′-ACCCTCTCTTCGCCTTCC-3′, GPX1 reverse 5′AGGACCAGCACCCATCTC-3′, *SEP15* forward 5′– GCCTGCTCCTCAGAGTCTC –3′ and *SEP15* reverse 5′–AAACATGAAAGAACAAACCAGAAG–3′. Digestion products were 158 bp, 232bo, 390 bp for *GPX1* and 360 bp, 198 bp, 162 bp *for SEP15*. Accuracy of Real Time PCR genotyping was checked by retyping and randomly selected samples (15 % form cases and controls). The compatibility of the results was 100 %.

#### BRCA1 mutation analysis

To exclude hereditary cancer cases attributed to mutations in high penetrance genes, we conducted genotyping for the two *BRCA1* mutations, which are most frequently observed among Polish population i.e.,: 5382insC and T300G (C61G) [19]. Both mutations were identified by the mismatch PCR and Restriction Fragment Analysis, respectively, using primer sequences as described in Additional file 1: Table S2. For both mutations, the PCR reactions were performed in a 20 μL volume, containing 100 ng template DNA, primers (1 μM each), 0.5 unit Taq polymerase (Qiagen, Hilden, Germany), dNTPs (150 μM each; Qiagen, Hilden, Germany), PCR reaction buffer (Qiagen, Hilden, Germany) and nuclease-free water. Reaction conditions covered initial DNA denaturation at 94 °C for 3 min, followed by 40 cycles: DNA denaturation at 94 °C for 45 s, annealing at 62 °C for 45 s and elongation at 72 °C for 1 min. The final extension was performed at 72 °C for 9 min. The PCR products were digested with endonucleases: *DDeI* (Promega, Madison, WI, USA) for 5382insC and *TaaI* (Fermentas, Burlington, Canada) for T300G mutation, according to the conditions described by suppliers. Digestion products were analyzed using electrophoretic technique in 2 % (w/v) agarose gel. Fragments’ length and interpretation of the results is indicated in Additional file 1: Table S2.

#### Lipid peroxidation

Plasma TBARS concentration was determined by the use of a spectrofluorometric method [20]. TBA-reactive compounds were extracted to butanol. The value of fluorescence of butanol layer was read at an excitation wavelength of λ = 525 nm and emission wavelength of λ = 547 nm, using the Perkin Elmer Luminescence Spectrometer LS50B (Norwalk, Ct, USA). Intraassay variation (CV) was 3.6 % (*n* = 8).

#### Glutathione peroxidase activity

Activity of GPx1 and GPx3 was determined in erythrocytes (GPx1) and plasma (GPx3) using the method of Paglia and Valentine [21] with t-butyl hydroperoxide as a substrate and following the rate of NADPH oxidation by the coupled reaction with glutathione reductase. The rate of decrease in the absorbance at 340 nm (being proportional to the GPx activity) was read using the Unicam UV4 UV/Vis spectrophotometer (Cambridge, UK). Intraassay variation (CV) was 2.7 % (*n* = 8) for GPx1 and 2.3 % (*n* = 7) for GPx3. The samples were analyzed in single measurements. The measurement was repeated whenever the value was out of the range.

#### Superoxide dismutase activity

Activity of SOD1 was determined in erythrocytes by the use of the method of Beauchamp and Fridovich [22], which relies on the inhibition by SOD of the reduction of Nitro Blue Tetrazolium (NBT) by xanthine and xanthine oxidase. Concentration of the reduced form of NBT was measured spectrophotometrically at a wavelength of λ = 540 nm, using the Unicam UV4 UV/Vis spectrophotometer (Cambridge, UK). Intraassay variation (CV) was 4.7 % (*n* = 8).

#### Ceruloplasmin activity

The oxidase activity of Cp was determined spectrophotometrically according to the method described by Sunderman and Nomoto [23], with a PPD (*p*-phenylenediamine) as a substrate. Absorbance of the oxidation product was read in Unicam UV4 UV/Vis spectrophotometer (Camridge, UK), at a wavelength of λ = 535 nm. The activity of Cp was expressed as the amount of product formed per minute per 1 L of plasma. Intraassay variation (CV) was 4.7 % (*n* = 5).

#### Selenium status

Plasma Se concentration was determined using the graphite furnace atomic absorption spectrophotometry with Zeeman background correction, in the Pye Unicam Solaar 989 QZ spectrophotometer (Cambridge, UK). Lyophilized human serum containing selenium at a concentration of 78 ng/L (Seronorm™, Nycomed Pharma AS, Norway) was used as a reference material for quality control and assurance. Additionally, the method was checked by participation in the interlaboratory comparison trials. Limit of detection for Se was 11 ng/mL and the precision calculated from 10 successive series of microelement determinations in the reference samples was 6.5 %.

### Statistical analysis

Normality for the data was evaluated with the Shapiro-Wilks test. The analysis of variance (ANOVA) or the Kruskal-Wallis test were used for the univariate analysis. Polymorphisms were entered individually into the ANCOVA model adjusting for clinical variables that could potentially affect the patient’s TBARS concentrations, including gene-disease interaction. Logistic regression analysis was used to evaluate the association of particular polymorphisms and their interactions with the disease status. Analysis of gene-gene interactions comprised *GPX1* x *SOD2* and *GPX1* x *SEPP1* as suggested by literature data [10, 24]. Other higher order interactions between genotypes were not fitted either in linear or logistic regressions due to the limited sample size. All the analyses were performed using STATISTICA 10 software package (Statsoft, Tulsa, OK, USA). All significance tests were two-sided and the statistical significance was established as *p* value less than 0.05.

## Results

Epidemiological and clinical characteristics of the study subjects are presented in Table 1. The patients with cancer did not differ significantly from the control group in terms of age, BMI, smoking status or menopausal status (Table 1). 80 % (109 women) of the cases were subjects diagnosed with invasive ductal carcinomas and 85 % (115 women) were before any clinical treatment. All the cases were negative for 5382insC and T300G (C61G) *BRCA1* mutations.

Distribution of *GPX1* (rs1050450), *GPX4* (rs713041), *SEPP1* (rs3877899), *SEP15* (rs5859) and *SOD2* (rs4880) genotypes in the study participants is presented in Table 2. Distribution of all alleles of the analyzed SNPs were in agreement with those expected under the Hardy-Weinberg equilibrium. Significant differences in allele frequencies were noted for the *GPX1* rs1050450 polymorphism, for which carrying at least one variant allele (*GPX1* Leu) was associated with a decreased risk of cancer both, in the univariate analysis and after adjustment for age, BMI, smoking status and menopausal status (Table 2). None of the 4 remaining polymorphisms showed any associations with the risk of breast cancer. The analysis of relevant gene-gene interactions was conducted for *GPX1* x *SOD2* and *GPX1* x *SEPP1* and did not reveal any significance (data not shown).

**Table 2:** Breast cancer risk associated with polymorphic variants in *GPX1*, *GPX4*, *SEPP1*, *SEP15* and *SOD2* genes

Polymorphism	Cases, *n* (%)	Controls, *n* (%)	OR crude (95%CI)	*p*	OR adjusted^a^ (95 % CI)	*p*
*GPX1* (rs1050450)						
Pro/Pro	73 (53.7)	75 (41.0)	1 (ref.)		1 (ref.)	
Leu/Leu	12 (8.8)	23 (12.6)	0.54 (0.25**–**1.16)	0.112	0.61 (0.28–1.34)	0.215
Pro/Leu + Leu/Leu	63 (46.3)	108 (59.0)	**0.60 (0.38–0.94)**	**0.026**	**0.61 (0.38–0.97)**	**0.035**
*GPX4* (rs713041)						
CC	44 (32.4)	65 (35.5)	1 (ref.)		1 (ref.)	
TT	26 (19.1)	28 (15.3)	1.37 (0.71–2.66)	0.345	1.31 (0.65–2.66)	0.445
CT + TT	92 (67.6)	118 (64.5)	1.15 (0.72–1.85)	0.556	1.12 (0.68–1.85)	0.644
*SEPP1* (rs3877899)^b^						
Ala/Ala	81 (60.4)	122 (66.7)	1 (ref.)		1 (ref.)	
Thr/Thr	9 (6.7)	6 (3.3)	2.26 (0.77–6.63)	0.136	2.57 (0.86–7.61)	0.087
Ala/Thr + Thr/Thr	53 (39.6)	61 (33.3)	1.31 (0.82–2.08)	0.255	1.38 (0.85–2.23)	0.192
*SEP15* (rs5859)						
GG	82 (60.3)	103 (56.3)	1 (ref.)		1 (ref.)	
AA	8 (5.9)	14 (7.7)	0.72 (0.28–1.80)	0.479	0.72 (0.27–1.91)	0.512
GA + AA	54 (39.7)	80 (43.7)	0.85 (0.54–1.33)	0.473	0.91 (0.57–1.46)	0.696
*SOD2* (rs4880)						
Ala/Ala	29 (21.3)	50 (27.3)	1 (ref.)		1 (ref.)	
Val/Val	32 (23.5)	41 (22.4)	1.34 (0.70–2,59)	0.371	1.54 (0.76–3.14)	0.227
Ala/Val + Val/Val	107 (78.7)	133 (72.7)	1.39 (0.82–2.35)	0.221	1.67 (0.95–2.96)	0.076

Significant *p* values are presented in bold

*OR* odds ratio, *95 % CI* 95 % confidence interval

^a^OR adjusted for age, BMI, menopausal status and smoking (ever, never)

^b^genotype status was unknown in the case of 2 individuals

Oxidative stress parameters in the cases and controls are presented in Table 3. Significantly higher TBARS levels and GPx1 activity (and *p* = 0.0003 and *p* = 0.0036, respectively) were observed in the women suffering from breast cancer as compared to the controls, whereas there were no differences in GPx3 and SOD activity. Also plasma Se concentration did not differ between the cases and controls, and accounted for 55.2 μg/L and 57.0 μg/L, respectively. Ceruloplasmin activity was significantly lower in the cases as compared to the controls (*p* = 0.0005; Table 3). Treatment status did not affect the levels and activities of the studied parameters, allowing us to retain the whole group of patients with cancer in further analyses (Additional file 1: Table S3).

**Table 3:** Oxidative stress parameters in the breast cancer cases and controls

Parameter	Cases (*n* = 136)	Controls (*n* = 183)	*p* ^*a*^ (vs controls)
GPx1 activity [U/g Hb]	22.3 ± 5.5 (11.1–35.0)	20.5 ± 4.7 (10.7–29.7)	**0.0036**
GPx3 activity [U/mL]	0.189 ± 0.037 (0.108–0.308)	0.191 ± 0.032 (0.125–0.297)	0.7491
SOD1 activity [U/mg Hb]	6.84 ± 1.24 (4.48–11.53)	6.90 ± 1.52 (3.03–10.91)	0.8590
Cp activity [g/L]	0.58 ± 0.18 (0.13–1.05)	0.66 ± 0.21 (0.31–1.75)	**0.0005**
TBARS concentration [μmol/L]	2.62 ± 0.96 (1.01–5.27)	2.24 ± 0.83 (1.00–5.90)	**0.0003**
Se concentration [μg/L]	55.2 ± 14.7 (23.2–99.9)	57.0 ± 11.8 (29.1–97.7)	0.1791

Data expressed as mean ± standard deviation and (range). Significant *p* values are presented in bold

^a^the Mann–Whitney test

**Fig. 1:**
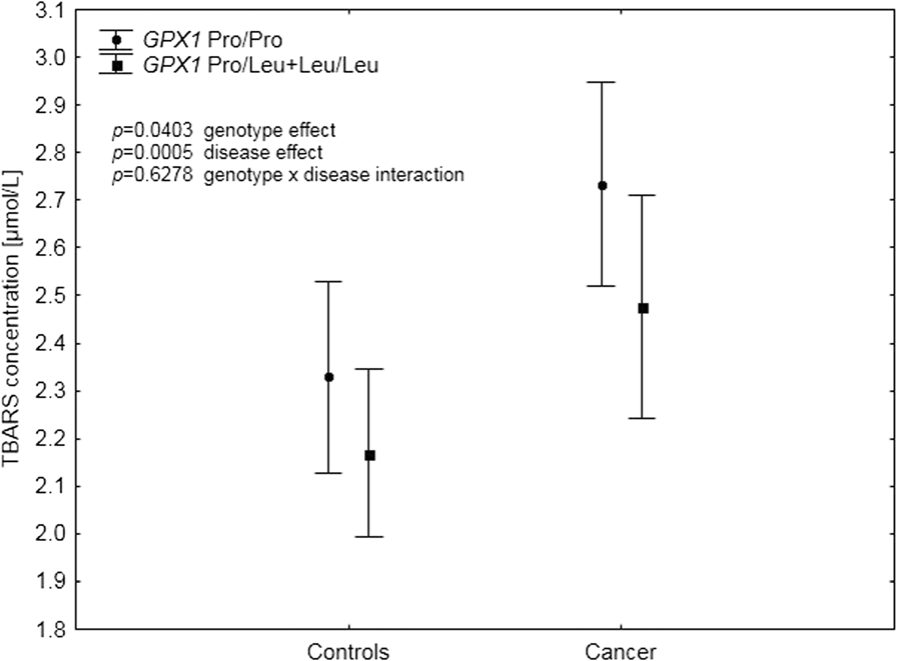
Additive effect of *GPX1* rs1050450 variants and the disease status on the plasma TBARS concentration. Data adjusted for age, BMI, current smoking and selenium

**Fig. 2:**
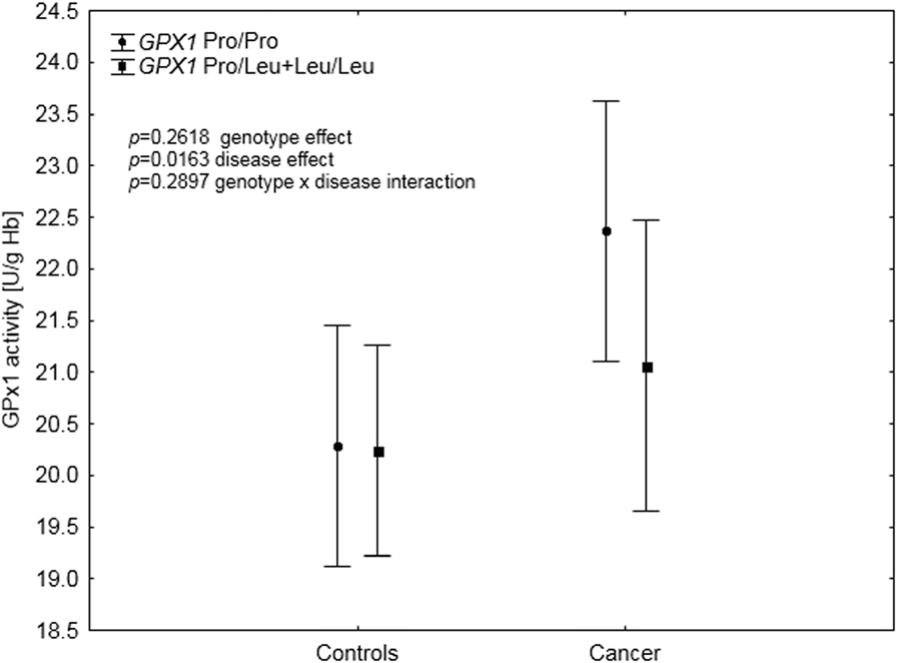
GPx1 activity depending on *GPX1* rs1050450 polymorphism and the disease status. Data adjusted for age, current smoking and selenium

**Fig. 3:**
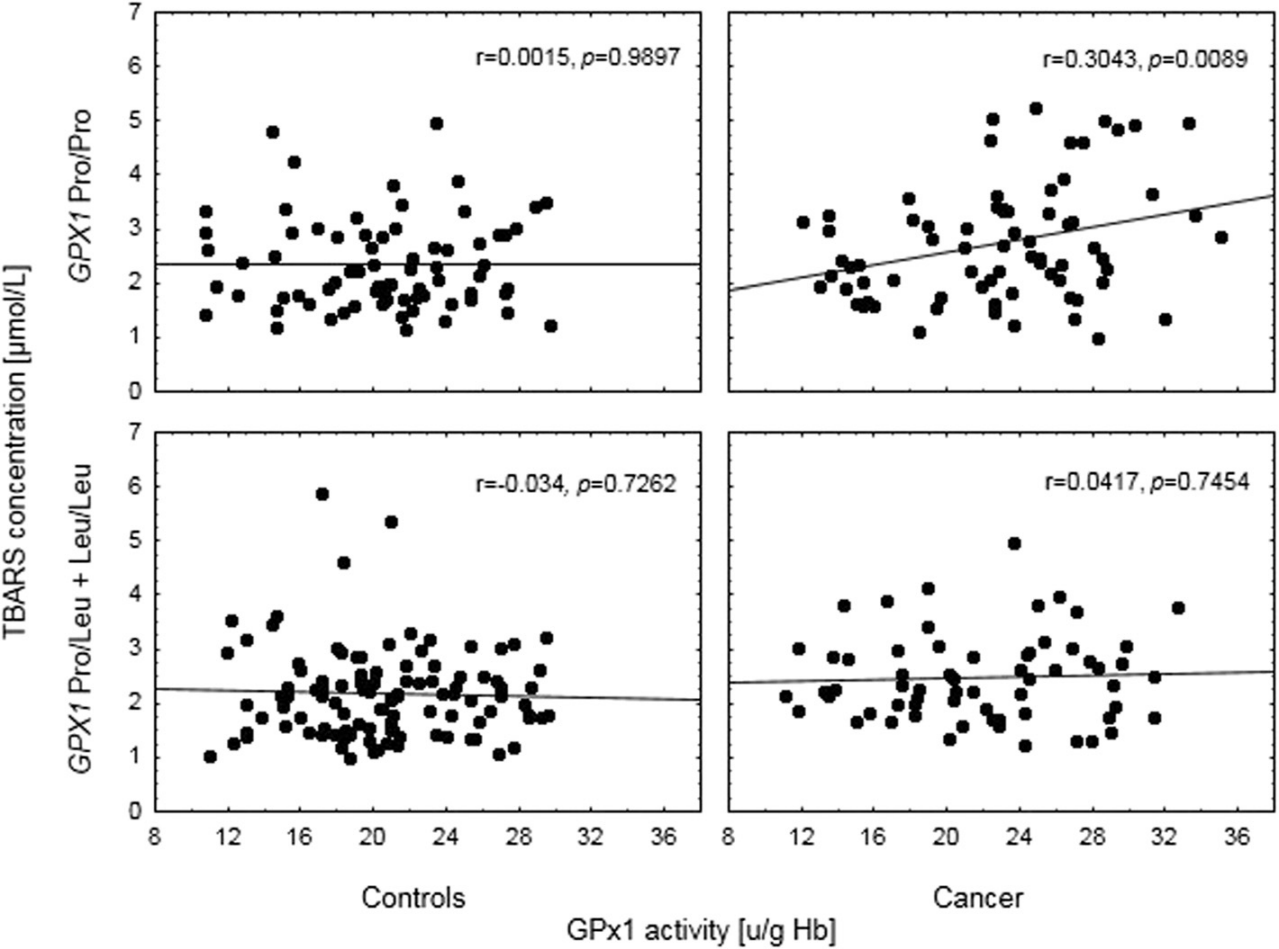
Correlation between TBARS and GPx1 activity depending on the disease status and the *GPX1* genotype. Correlation coefficients in the breast cancer cases

Table 4 presents data on lipid peroxidation in the study group analyzed with respect to different genotypes. Carrying the polymorphic variant of the *GPX1* gene was shown to significantly affect plasma TBARS concentrations, with wild-type homozygotes, having higher levels than the individuals with at least one polymorphic allele (*p* = 0.0320; Table 4). There were no differences in TBARS levels with respect to other genotypes. Further investigation of the observed association between *GPX1* polymorphism and TBARS was conducted with respect to disease status, in a multivariate regression model with age, BMI and smoking status (Table 5). Results showed that the effect of both malignancy and *GPX1* genotypes on TBARS levels is additive rather than conditional (Fig. 1). The patients with cancer showed TBARS concentrations higher by 0.18 μmol/L, than those observed in the controls. At the same time, carrying at least one polymorphic allele at *GPX1* was associated with TBARS levels lower by 0.10 μmol/L. This resulted in Pro/Pro homozygotes with cancer having the highest TBARS levels among all the 4 groups (2.74 95 % CI 2.53–2.95). Following that, we investigated whether this association would be linked directly through GPx1 activity. TBARS levels showed a positive correlation with GPx1 activity, which was close to statistical significance (*r* = 0.1056; *p* = 0.0596). Neither Cp (*r* = 0.0016; *p* = 0.9778), SOD1 (*r* = −0.0262; *p* = 0.6411), GPx3 (*r* = 0.0805; *p* = 0.1514) activities nor Se concentration (*r* = 0.0141; *p* = 0.801) showed such associations. However, we did not observe any direct associations between GPx1 activity and the *GPX1* rs1050450 polymorphic allele presence in the univariate analysis (*p* = 0.2669) or after adjustment for age, BMI, smoking status and Se status (beta = − 0.07; *p* = 0.2618, Table 5). Interaction between the presence of malignancy (the disease status) and *GPX1* genotype was not significant (*p* = 0.2897), although Pro/Pro homozygotes with cancer showed GPx1 activity higher by 1.5–2.0 U/g Hb than all the other variants (Table 5, Fig. 2). Given the significant impact of the disease and genotype at rs1050450 on TBARS and the apparent correlation between GPx1 activity and TBARS, we evaluated whether the latter effect is in fact group-dependent (Fig. 3). Among the individuals with the Pro/Pro genotype, the correlation between TBARS concentration and GPx1 activity was positive and significant (*r* = 0.3043; *p* = 0.0089) but it was absent in the cancer patients who had one or two polymorphic *GPX1* Leu alleles (*r* = 0.0417; *p* = 0.7454). This effect was completely absent in the Pro/Pro controls who obviously had the lowest range of both TBARS and GPx1 (*r* = −0.0015; *p* = 0.9897) as well as in the controls positive for the Leu allele (*r* = −0.034; *p* = 0.7262).

**Table 4:** Plasma TBARS concentration in all the individuals (cases and controls), data stratified according to the genotype

Polymorphism	Genotype	N	TBARS concentration [μmol/L]	*p* ^*a*^ (ANOVA)	*p* ^*b*^ (vs wild type homozygote)
*GPX1* (rs1050450)	Pro/Pro	148	2.32 (1.80–3.09)	0.0527	
	Pro/Leu	136	2.15 (1.69–2.64)		
	Leu/Leu	35	2.29 (1.75–2.94)		
	Pro/Leu + Leu/Leu	171	2.18 (1.70–2.73)		**0.0320**
*GPX4* (rs713041)	CC	109	2.23 (1.82–2.85)	0.4266	
	CT	156	2.21 (1.75–2.89)		
	TT	54	2.38 (1.86–3.07)		
	CT + TT	210	2.34 (1.76–2.94)		0.3237
*SEPP1* (rs3877899)^c^	Ala/Ala	203	2.26 (1.76–2.94)	0.9393	
	Ala/Thr	99	2.21 (1.74–2.87)		
	Thr/Thr	15	2.05 (1.65–2.93)		
	Ala/Thr + Thr/Thr	114	2.19 (1.74–2.87)		0.7802
*SEP15* (rs5859)	GG	185	2.26 (1.78–2.93)	0.8028	
	GA	112	2.22 (1.69–2.92)		
	AA	22	2.03 (1.75–2.86)		
	GA + AA	134	2.21 (1.73–2.89)		0.5357
*SOD2* (rs4880)	Ala/Ala	79	2.32 (178–3.00)	0.8902	
	Ala/Val	167	2.23 (1.76–2.87)		
	Val/Val	73	2.22 (1.69–2.92)		
	Ala/Val + Val/Val	240	2.23 (1.74–2.88)		0.6891

Data expressed as median values and (25 and 75 % percentiles). Significant *p* values are presented in bold

^a^the Kruskal-Wallis test

^b^the Mann–Whitney test

^c^genotype status was unknown in the case of 2 individuals

**Table 5:** Multivariate regression model for the factors associated with TBARS concentration and GPx1 activity

	TBARS - Beta (ß)	TBARS – *p*	GPx1 – Beta (ß)	GPx1– *p*
Age	**0.1281**	**0.0277**	**0.1378**	**0.0230**
BMI	0.0986	0.0926	−0.0192	0.7523
Smoking status	−0.0227	0.6817	**−0.1126**	**0.0491**
Selenium	^a^	^a^	−0.0877	0.1212
*GPX1* Pro/Pro vs Pro/Leu + Leu/Leu	**−0.1150**	**0.0403**	−0.0652	0.2618
Disease status	**0.1940**	**0.0005**	**0.1383**	**0.0163**
*GPX1* Pro/Pro vs Pro/Leu + Leu/Leu ^a^ Disease status	0.0269	0.6278	0.0610	0.2897

Significant values are presented in bold

^a^factor not included in the model

## Discussion

The role of lipid peroxidation in breast cancer remains not fully elucidated. The main focus of this study was to investigate whether lipid peroxidation in breast cancer subjects is associated with genetic polymorphism of antioxidant enzymes. In addition, we analyzed the risk of breast cancer in association with selected gene variants as well as with Se status.

### Lipid peroxidation in breast cancer – link with GPX1 polymorphism and GPx1 activity

We observed a higher concentration of TBARS in plasma of the breast cancer cases as compared to the healthy women. This observation is consistent with the general observation of increased lipid peroxidation in breast cancer. Numerous studies have shown increased levels of different markers of lipid peroxidation (TBARS or specific aldehydes like malondialdehyde, 8-F2 isoprostanes or 4-hydroksynonenal) in plasma, serum, urine and also, in some cases, in cancer tissue of the women suffering from breast cancer [24–30]. In our study, increased plasma lipid peroxidation in cancer subjects was accompanied by the increased activity of GPx1 (Table 3), supporting other findings on the altered antioxidant homeostasis in breast cancer [24–30]. Interestingly, we observed a positive correlation between plasma TBARS concentration and GPx1 activity measured in blood erythrocytes of the breast cancer subjects. So far, few authors have investigated the correlation between lipid peroxidation and activity of antioxidant enzymes in cancer patients, focusing rather on the differences between the selected parameters. Interestingly, the correlation between plasma lipid peroxidation and erythrocyte glutathione peroxidase seems to depend on health status, being for example positive in healthy subjects and negative in the subjects undergoing chronic hemodialysis [31, 32]. Tas et al. have investigated such a relationship in breast cancer patients, showing no correlation between MDA levels and GPx1 activity in cancer tissue though both parameters were significantly increased as compared to benign tumors [30]. However, the positive correlation has been found in the same study between MDA levels and the activity of Cat (the enzyme which similarly as GPx1, catalyzes the reduction of hydrogen peroxide).

Additionally, in our study we observed that TBARS concentration was associated with *GPX1* rs1050450 polymorphism (Tables 4 and 5). This SNP is linked to the amino acid substitution, from proline (Pro) to leucine (Leu), and this change was shown to affect GPx1 activity, with the polymorphic variant (Leu) being less responsive to Se as observed *in vitro*, in human breast cancer cells (MCF-7) [33]. In our previous observational study we have found that the correlation between GPx1 activity and plasma Se concentration in humans seems to depend on *GPX1* polymorphism, being significant only among individuals carrying at least one Pro allele [34]. In this study we failed to indicate a significant effect of *GPX1* polymorphism on GPx1 activity in the whole group. However, the SNP effect seemed to be preserved among cancer cases, with Pro/Pro cancer homozygotes having the highest GPx1 activity as compared to other groups. Furthermore, only in this genotype group there was a significant and positive correlation between GPx1 activity and lipid peroxidation. This observation suggests that *GPX1* rs1050450 polymorphism may actually determine not only the response of GPx1 activity to Se supplementation, but also its response to lipid peroxidation (and generally oxidative stress), at least in breast cancer subjects.

Since lipid peroxidation products have been recently recognized as a therapeutic target in cancer, the possible relationship between lipid peroxidation and *GPX1* polymorphism could have a potential role in breast cancer treatment. Notably it has been already observed that products of lipid peroxidation may modulate processes crucial in the breast cancer survival [2]. Thus it may be speculated that *GPX1* polymorphism may affect breast cancer treatment via modulating lipid peroxidation. It remains to be elucidated, which genotype would be more favorable in terms of a better therapy outcome. One could expect the individuals with *GPX1* Pro/Pro to be less vulnerable to prooxidant effects of the treatment due to a higher antioxidant response under a permanent stress condition. The rationale for this assumption is supported by studies which indicated that increased GPx1 activity was associated with anticancer drug resistance [35, 36]. Possible role of *GPX1* polymorphism in modifying the response to anticancer therapy has already been suggested by Zhao et al. These authors conducted a prospective study on 224 patients with bladder cancer and observed that individuals possessing Pro/Pro genotype had shorter recurrence–free survival as compared to those with at least one variant (Leu) allele [37]. A significant protective effect of Leu alleles was observed only among the whites (*n* = 202) with a hazard ratio (HR) of 0.63; 95 % CI 0.42–0.96. Interestingly, after data stratification according to sex, the effect was preserved only among women. The authors of this study suggest that the unexpectedly observed protective effect of Leu allele may be explained by the fact that the patients with a reduced activity of ROS scavenging enzymes may have better prognosis after cancer treatment as most of the therapies (immunotherapy, chemotherapy, radiotherapy) are based on ROS generation [37].

### Breast cancer risk associated with SNPs in the antioxidant enzymes

Breast cancer risk was significantly associated with *GPX1* rs1050450 polymorphism in this study. In this study we observed that carrying Leu variant was associated with a significant 40 % decrease in the risk (Table 2). These findings are not consistent with the results of the recent study by Meplan et al., in which *GPX1* Leu/Leu genotype has been linked to a significantly increased risk of breast cancer (adjusted OR = 1.88; 95%CI 1.08–3.28) [38]. The results of earlier case control studies on breast cancer risk and *GPX1* rs1050450 polymorphism are also inconsistent between each other, showing lack of any associations or the increased risk linked to the carriage of the variant allele [8, 39–41]. Recent meta-analysis performed by Hu et al., which covered 5509 breast cancer cases and 6542 controls from 6 case control studies, has not revealed any association among the whites and has suggested that the polymorphic variant may increase the risk only among Africans [42]. However, the meta-analysis has not considered histopathological type of breast cancer and it is likely that since there are different risk factors for ductal and non-ductal breast cancer, the effects of SNPs may also vary considerably [43]. Notably, the significant association for *GPX1* polymorphism in the mentioned study by Meplan et al., has been restricted only to the non-ductal cancers [38]. Nevertheless, we did not expect to find significant odds ratios in this study (it was not the main aim of the study) due to the small sample size, and there is a high probability that the observed effect of *GPX1* polymorphism could occur by chance. However, it has been the first such a study regarding sporadic breast cancer risk and *GPX1* polymorphism conducted among Polish women, and considering the fact that similar protective effect of *GPX1* Leu variant has been found in Polish population also in the case of lung and laryngeal cancers [44], the presented results deserve further investigation. For other investigated SNPs: rs713041 (*GPX4*), rs3877899 (*SEPP1*), rs5859 (*SEP15*) and rs4880 (*SOD2*), we failed to find any associations with the breast cancer risk. Similarly, we did not observe any significant gene-gene interactions of potential interest as suggested by other studies, including *GPX1* x *SOD2* and *GPX1* x *SEPP1* [8, 38].

### Breast cancer risk and Se status

In the study presented here, plasma Se concentration was relatively low (29.1–99.9 μg/L; Table 3) in all the study participants (being consistent with the fact of low dietary Se intake in Polish population [45]). However, it was not associated with the breast cancer risk. In general, the association between breast cancer and Se status remains controversial. Some case control studies have indicated the increased risk linked to the low dietary intake of the element, its low concentration in plasma/serum or low content in toe nails [46, 47] but no such association has been found in other studies, both with a retrospective [48] and a prospective approach [49–52]. Interestingly, the authors of one case control study, in which serum Se has been found to be lower in the breast cancer women (*n* = 200) as compared to the healthy controls (*n* = 200), have concluded that altered Se status was a consequence rather than a cause of cancer [46]. Potentially protective activity of Se compounds against breast cancer has been suggested on the basis of *in vitro* and *in vivo* observations, indicating for the regulatory activity of Se on estrogen receptors expression [53–55]. In the light of epidemiological data however, including our study, link between Se and breast cancer remains still elusive.

### Limitations of the study

Results of this study could have been biased by non-random selection of the control subjects. It should be noted that the study presented here was not a typical case control study. Thus, we were not able to assign the same confounders to both groups (cancer cases and control subjects) and cannot rule out that the observed associations were not influenced by potential confounding factors, not controlled for in the study (as for example diet or supplements use). Nevertheless, both groups were residents of the same area, and were not different in terms of age, BMI, smoking status and menopausal status. It should be also appreciated that all the subjects enrolled in the control group had negative screening mammograms and this information was crucial in the assessment of biochemical processes linked to breast cancer. Another weakness of the study concerns relatively small sample size, which limited the possibility to include more potentially important modifiers of both GPx1 activity and TBARS levels, such as for example ER status. Finally, this study lacked data on patients’ survival, which obviously would give further insights into the clinical significance of the observed association between lipid peroxidation and *GPX1* polymorphism.

## Conclusions

Up to date, no studies have been conducted on the association between individual genetic background and markers of prooxidative effects in breast cancer. The results of this study suggest that *GPX1* polymorphism may be an important factor that modifies oxidative stress response in breast cancer. The potential link may have great significance in terms of potential implication in tumor progression or treatment thus these findings, if replicated elsewhere, require further investigation.

## Additional file

Additional file 1: Table S1. Functional SNPs selected for the study. **Table S2**. Restriction fragment analysis for BRCA1 mutations. **Table S3**. Oxidative stress parameters in breast cancer cases according to treatment. (DOCX 31 kb)

## Abbreviations

BMI: Body mass index
*BRCA1*: Breast cancer 1, early onset (gene)
*BRCA2*: Breast cancer 2, early onset (gene)
Cat: Catalase
Cp: Ceruloplasmin
*GPX1*: Cytosolic glutathione peroxidase (gene)
GPx1: Cytosolic glutathione peroxidase
GPx2: Gastrointestinal glutathione peroxidase
*GPX3*: Plasma glutathione peroxidase (gene)
GPx3: Plasma glutathione peroxidase
*GPX4*: Phospholipid hydroperoxide glutathione peroxidase (gene)
GPx4: phospholipid hydroperoxide glutathione peroxidase
HR: Hazard ratio
HRM: High resolution melting
Leu: Leucine
MDA: Malondialdehyde
OR: Odds ratio
PCR: Polymerase chain reaction
Pro: Proline
ROS: Reactive oxygen species
Se: Selenium
*SEP15*: 15-kDa selenoprotein (gene)
*SEPP1*: Selenoprotein P (gene)
SelP: Selenoprotein P
SNP: Single nucleotide polymorphism
*SOD2*: Mitochondrial superoxide dismutase (gene)
SOD1: Cytosolic superoxide dismutase
SOD2: Mitochondrial superoxide dismutase
SOD3: Extracellular superoxide disumutase
TBARS: Thiobarbituric acid reactive substances
