# Supplementary material for: Lipid peroxidation and glutathione peroxidase activity relationship in breast cancer depends on functional polymorphism of GPX1
Source: BMC Cancer. 2015 Oct 7;15:657. doi: 10.1186/s12885-015-1680-4 (PMC4597452; doi:10.1186/s12885-015-1680-4)
Supplement: Additional file 1: Table S1. — Functional SNPs selected for the study. Table S2. Restriction fragment analysis for BRCA1 mutations. Table S3. Oxidative stress parameters in breast cancer cases according to treatment. (DOCX 31 kb) [file 12885_2015_1680_MOESM1_ESM.docx]

**Additional file 1**

**Table S1.** Functional SNPs selected for the study.

| **Gene (locus)** | **Encoded protein** | **dbSNP** | **Allele change** | **Function** | **Functional effects** |
| --- | --- | --- | --- | --- | --- |
| *GPX1*  (3p21.3) | Cytosolic glutathione peroxidase | rs1050450 | C/T | Missense (Pro200Leu) | Leu (T) variant was associated with lower induction of GPx1 activity in the response to Se supplementation in transfected human breast carcinoma cells [1]. |
| *GPX4*  (19p13.3) | Phospholipid glutathione peroxidase | rs713041 | T/C | Synonymous (Leu200Leu) | C variant was associated with a higher concentration of lymphocyte 5- lipoxygenase metabolites (Vilette et al., 2002). The SNP also affected GPx4 levels in lymphocytes under low Se supply  (Meplan et al., 2008) and altered selenoprotein expression patterns in transfected human epithelial colorectal adenocarcinoma cells [2]. |
| *SEPP1*  (5q31) | Selenoprotein P | rs3877899 | G/A | Missense (Ala234Thr) | The SNP was shown to influence plasma Sepp1 isoform pattern in human Se supplementation trial [3]. |
| *SEP15*  (1p31) | 15-kDa selenoprotein | rs5859 | G/A | UTR-3 | Gene reporter assay showed that the SNP affected function of specific mRNA stem loop structure (called SECIS) within *SEP15* gene [4]. |
| *SOD2*  (6q25.3) | Superoxide dismutase 2 (mitochondrial) | rs4880 | T/C | Missense (Val16Ala) | Ala (T) variant was associated with a higher SOD2 activity as shown in transfected human hepatoma cells [5] |

**Table S2.** Restriction fragment analysis for *BRCA1* mutations.

| **Mutation** | **Forward primer** | **Reverse primer** | **Restriction enzyme** | **Length (bp)** | |
| --- | --- | --- | --- | --- | --- |
|  |  |  |  | **Normal** | **Mutant** |
| **5382insC** | 5’-CCAAAGCGAGCAAGAGAATCCC-3’ | 5’-GGGAATCCAAATTACACAGC-3’ | *Dde*I | 214-36-20 | 235-36 |
| **T300G (C61G)** | 5’-CTCTTAAGGGCAGTTGTGAG-3’ | 5’-ATGGTTTTATAGGAACGCTATG-3’ | *Taa*I | 154-74-50 | 228-50 |

**Table S3.** Oxidative stress parameters in breast cancer cases according to treatment.

|  | **Cases** | | ***p- value*** |
| --- | --- | --- | --- |
| **Parameter** | **Untreated**  **(n = 115)** | **Treated**  **(n = 21)** |  |
| **GPx1 activity**  **[U/g Hb]** | 22.4 ± 5.4  (11.1-35.0) | 21.6 ± 6.6  (12.1-33.7) | 0.556^a^ |
| **GPx3 activity**  **[U/mL]** | 0.189 ± 0.038  (0.108-0.308) | 0.191 ± 0.000  (0.136-0.252) | 0.778^a^ |
| **SOD1 activity**  **[U/mg Hb]** | 6.92 ± 1.23  (4.48-11.53) | 6.41 ± 1.21  (4.93-9.00) | 0.090^b^ |
| **Cp activity**  **[g/L]** | 0.58 ± 0.17  (0.13-1.05) | 0.59 ± 0.21  (0.30-1.03) | 0.783^a^ |
| **TBARS**  **[µmol/L]** | 2.62 ± 0.99  (1.01-5.27) | 2.62 ± 0.79  (1.65-4.60) | 0.775^b^ |
| **Se**  **[µg/L]** | 55.3 ± 14.4  (23.2-99.9) | 54.7 ± 16.4  26.5-86.4) | 0.882^a^ |

Data expressed as mean ± standard deviation (range),

^a^ – Student’s t test, ^b^ – Mann-Whitney test

**Supplementary references**

1. Hu YJ, Diamond AM: **Role of glutathione peroxidase 1 in breast cancer: loss of heterozygosity and allelic differences in the response to selenium.** *Cancer Res* 2003, **63**:3347–51.

2. Gautrey H, Nicol F, Sneddon AA, Hall J, Hesketh J: **A T/C polymorphism in the GPX4 3’UTR affects the selenoprotein expression pattern and cell viability in transfected Caco-2 cells.** *Biochim Biophys Acta* 2011, **1810**:584–91.

3. Méplan C, Nicol F, Burtle BT, Crosley LK, Arthur JR, Mathers JC, Hesketh JE: **Relative abundance of selenoprotein P isoforms in human plasma depends on genotype, se intake, and cancer status.** *Antioxid Redox Signal* 2009, **11**:2631–40.

4. Hu YJ, Korotkov K V, Mehta R, Hatfield DL, Rotimi CN, Luke A, Prewitt TE, Cooper RS, Stock W, Vokes EE, Dolan ME, Gladyshev VN, Diamond AM: **Distribution and functional consequences of nucleotide polymorphisms in the 3’-untranslated region of the human Sep15 gene.** *Cancer Res* 2001, **61**:2307–10.

5. Sutton A, Imbert A, Igoudjil A, Descatoire V, Cazanave S, Pessayre D, Degoul F: **The manganese superoxide dismutase Ala16Val dimorphism modulates both mitochondrial import and mRNA stability.** *Pharmacogenet Genomics* 2005, **15**:311–9.
